# Functionally-focused algorithmic analysis of high resolution microarray-CGH genomic landscapes demonstrates comparable genomic copy number aberrations in MSI and MSS sporadic colorectal cancer

**DOI:** 10.1371/journal.pone.0171690

**Published:** 2017-02-23

**Authors:** Hamad Ali, Milad S. Bitar, Ashraf Al Madhoun, Makia Marafie, Fahd Al-Mulla

**Affiliations:** 1 Department of Medical Laboratory Sciences, Faculty of Allied Health Sciences, Kuwait University, Jabriya, Kuwait; 2 Research Division, Immunology Unit, Dasman Diabetes Institute (DDI), Dasman, Kuwait; 3 Department of Pharmacology & Toxicology, Faculty of Medicine, Kuwait University, Jabriya, Kuwait; 4 Kuwait Medical Genetics Center, Kuwait City, Kuwait; 5 Molecular Pathology Unit, Department of Pathology, Faculty of Medicine, Kuwait University, Jabriya, Kuwait; 6 Research Division, Genomics Unit, Dasman Diabetes Institute (DDI), Dasman, Kuwait; Howard University, UNITED STATES

## Abstract

Array-based comparative genomic hybridization (aCGH) emerged as a powerful technology for studying copy number variations at higher resolution in many cancers including colorectal cancer. However, the lack of standardized systematic protocols including bioinformatic algorithms to obtain and analyze genomic data resulted in significant variation in the reported copy number aberration (CNA) data. Here, we present genomic aCGH data obtained using highly stringent and functionally relevant statistical algorithms from 116 well-defined microsatellites instable (MSI) and microsatellite stable (MSS) colorectal cancers. We utilized aCGH to characterize genomic CNAs in 116 well-defined sets of colorectal cancer (CRC) cases. We further applied the significance testing for aberrant copy number (STAC) and Genomic Identification of Significant Targets in Cancer (GISTIC) algorithms to identify functionally relevant (nonrandom) chromosomal aberrations in the analyzed colorectal cancer samples. Our results produced high resolution genomic landscapes of both, MSI and MSS sporadic CRC. We found that CNAs in MSI and MSS CRCs are heterogeneous in nature but may be divided into 3 distinct genomic patterns. Moreover, we show that although CNAs in MSI and MSS CRCs differ with respect to their size, number and chromosomal distribution, the functional copy number aberrations obtained from MSI and MSS CRCs were in fact comparable but not identical. These unifying CNAs were verified by MLPA tumor-loss gene panel, which spans 15 different chromosomal locations and contains 50 probes for at least 20 tumor suppressor genes. Consistently, deletion/amplification in these frequently cancer altered genes were identical in MSS and MSI CRCs. Our results suggest that MSI and MSS copy number aberrations driving CRC may be functionally comparable.

## Introduction

Comparative genomics have been extensively used to identify DNA copy number variations in cancer. At the chromosomal level, Mertens et al. assessed the distribution of chromosomal gains and losses in published karyotypes from 11 tumour types including 333 cases of colorectal carcinomas (CRCs) [1]. In CRC, recurrent gains in chromosomes 7, 8q, 13 and 20; and losses of lp, 5q, 8p, 14p, 17p, 18 and 22 were found [2, 3]. Later, metaphase-based comparative genomic hybridization (m-CGH), a technique of about 5 million bases resolution, was utilized to decipher chromosomal copy number changes in CRC progression. A plethora of studies used m-CGH to identify chromosomal imbalances in CRC, which included gain of chromosomes 1, 13 and 20 and chromosome arms 7p and 8q, whereas chromosome 4 and chromosome arms 8p and 10q were frequently deleted [2–7]. However, the large size of genomic aberrations identified by these low resolution techniques, which may contain hundreds of genes, prohibited the precise identification of DNA stretches involved in CRC progression [8–12]. In 2004, array-based comparative genomic hybridization (aCGH) emerged as a more promising technology for studying copy number variations at higher resolutions even from formalin-fixed paraffin-embedded (FFPE) archived material [13–17]. Bacterial artificial chromosome or BAC-based microarrays have a resolution of 1 million bases, while oligonucleotide-based microarrays have a much higher resolution of 8.9 KB overall median probe spacing (7.4 KB in Refseq genes) with 44,000 to 4.5 million probes dotted on slides, many of which densely cover all known or possible human genes [8]. Oligonucleotide-based aCGH is now capable of identifying copy number alterations in few thousands of bases or smaller [18–20].

This powerful technology, however, comes with its own limitations. For example, similar or even the same samples performed on different platforms may yield significantly different results. Moreover, the lack of standardized bioinformatic algorithms or analytical methods used to detect genomic aberrations complicates conclusions even further. These are compounded by the inherent heterogeneous nature of cancer evolution [21]. Seldom do aCGH-based studies account for such biologically confounding variables. Consequently, previously published colorectal cancer-related aCGH studies have yielded a high level of discordance in the reported genomic aberrations of colorectal cancer [20, 22, 23]. In addition, traditional means of classifying the importance of cancer-related copy number aberrations (CNAs) include the frequency of their occurrence in different patients. However, cancer genomes are highly complex and frequently harbor random 'passenger' CNAs that are of no functional significance [24]. To alleviate interference from those non-random CNAs, a systematic method, termed Genomic Identification of Significant Targets in Cancer (GISTIC) was recently developed and used in identifying biologically significant CNAs in several cancer types. The GISTIC algorithm determines a 'G' score based on the frequency and amplitude of the gains and losses. By giving more weight to high copy gains and homozygous losses (amplitude), the GISTIC algorithm argues that such aberrations may be more functionally relevant to the successful evolution of the cancer genome [25–27].

Here, we have utilized genomic high-density oligonucleotide-based microarrays to identify CNAs in well-defined colorectal cancers. In addition, we used the GISTIC algorithm to identify 'driver' chromosomal aberrations in colorectal cancer. We identified 3 distinct CNA patterns in CRCs and show that although CNAs in MSI and MSS CRCs differ with respect to their size, number and chromosomal distribution, the evolutionary and biologically relevant driver mutations of MSI and MSS CRC are not as dissimilar with respect to non-randomcopy number aberrations as traditional methods have previously suggested [15,28].

## Materials and methods

### CRC samples

A total of 116 CRC patients were recruited for this study. The study’s protocols were approved by by the Health Sciences Center (HSC) and Kuwait Institute for Medical Specialization (KIMS) joint committee for the protection of human subjects in research. Written informed consent was obtained from all patients before their inclusion in the study. DNA extracted from formalin-fixed paraffin-embedded (FFPE) tissues from 96 patients with sporadic early stage II CRC were used for genomic profiling. Genomic DNA was isolated from microdissected FFPE CRC tissues as described previously [29].

### aCGH for FFPE samples

#### a. Labeling of genomic DNA

Human Genome CGH Microarray 244A slides (Agilent Technologies, CA, USA) were used for FFPE extracted DNA samples. We followed the protocol described in [30, 31]. A total of 2.5 μg sex matched control DNA (Promega, WI, USA) was fragmented by sonication. The FFPE DNA was fragmented only if there was any large molecular weight DNA. About 500 ng of the fragmented samples were then run on 1.5% agarose gel for 1 hour to check the extent of fragmentation of the DNA. Once fragmentation was deemed appropriate, 2 μg of the control DNA was labeled with Cy3 (Agilent technologies, CA, USA) and 2μg of FFPE DNA with Cy5 (Agilent technologies, CA, USA) for 30 minutes at 85°C. After labeling the DNA was purified using KREA pure columns (Agilent technologies, CA, USA). The samples were then measured on a Nanodrop and Degree of Labeling (DOL) was calculated according to the following formula;

DegreeofLabeling=(340xpmol/μlofdye)/(ng/lμofGenomicDNAx1000)

The samples were hybridized onto microarray slides only if the DOL was between 1.5–2.5%.

#### b. Hybridization of labeled DNA

Appropriate volumes of Human Cot-I DNA (Invitrogen, CA, USA), 10X Blocking Agent (Agilent Technologies) and 2X Hybridization buffer (Agilent Technologies) were added to the paired Cy5 and Cy3 labeled DNA and the hybridization mix was mixed by pipetting gently. The Hybridization cocktail was then incubated at 95°C for 3 minutes, then immediately followed by 30 minutes at 37°C. The tubes were spun to collect the samples and an appropriate volume of Agilent-CGH block buffer (Agilent technologies, CA, USA) was added to the hybridization cocktail. The samples were mixed gently and centrifuged for collection. We dispensed 490 μl of the hybridization cocktail onto a clean gasket slide, which was already placed into a hybridization chamber. A microarray slide was placed active side down onto the gasket. The chamber was assembled and incubated in the hybridization rotating oven (Agilent technologies, CA, USA) for 40 hours at 60°C and 20 rpm.

#### c. Washing of the microarray slide

The hybridization chamber was disassembled carefully and the microarray slide sandwich was completely submerged into wash buffer 1 (Agilent technologies, CA, USA) at room temperature. The slides were then gently pried open using a pair of forceps and the gasket was allowed to drop to the bottom of the jar. The microarray slide was quickly transferred to a slide rack submerged in wash buffer 1 and incubated for 5 minutes. Then the rack was transferred to the next dish containing wash buffer 2 (Agilent technologies, CA, USA) at 37°C for 1 minute. The slide rack was then transferred to a dish containing Acetonitrile for 1 minute followed by 30 seconds in Stabilization solution (Agilent technologies). The rack was removed carefully in order to minimize the number of droplets on the slide. The slides were scanned immediately on an Agilent scanner.

#### d. Data analysis of the sample

Scanned images were imported; background subtracted and normalized using Feature extraction software version 10.7.1.1 (Agilent Technologies, CA, USA). The feature extraction software generates a quality Control Report which helps determine the quality of the aCGH. Quality Control metrics such as derivative of log ratio spread (DLRS), background noise (BG noise), signal intensity, reproducibility and signal to noise ratio are generated in the QC report.DLR Spread is defined as the spread of the Log Ratio differences between consecutive probes along all chromosomes. It is the most important metric as it gives us the ability to measure noise of the log ratio independent from the number and severity of aberrations found, making it instrumental in assessing the overall quality of each microarray experiment. If the DLR Spread value was higher than 0.5, the cases were excluded from further analysis. The text files representing data ratio points log2 of test/control ratios were imported to Nexus software (Biodiscovery, CA, U.S.A). Quality values ranged between 0.05–0.4, which are excellent values given the degraded nature of the samples. To minimize false positive calls and random CNV variations, Fast Adaptive State Segmentation Technique (FASST2) with a stringent significance threshold of 5.0E-6 was used to determine copy number aberrations. Moreover, we utilized two algorithms to more accurately reflect functional CNVs and separate them from bystander genomic aberrations. The first termed Significance Testing for Aberrant Copy number (STAC) algorithm, and the second is a systematic method termed Genomic Identification of Significant Targets in Cancer (GISTIC) o identify biologically significant copy number aberrations in these samples. Both algorithms were calculated using Nexus software version 8 (Biodiscovery, El Segundo CA, USA)

### Multiplex Ligation-dependent Probe Amplification (MLPA)

DNA was diluted to a working stock concentration of 50 ng/μl. A total of 250 ng (5μl) of DNA was aliquoted into sterile 0.2 ml tubes and denatured and then cooled to 25°C in a thermal cycler. A master mix containing hybridization components supplied as part of the kit (SALSA MLPA probemix P294-B1 Tumour-Loss from MRC Holland, Amsterdam, Netherlands) was prepared and added to the samples at 25°C and the reaction was mixed by pipetting. The hybridization reaction was carried out according to the manufacturer’s instructions and the samples were incubated overnight at 60°C. A ligase buffer mix was prepared with reagents supplied with the kit and ligation was carried out with Liagse-65. Following ligation, a PCR master mix was prepared using SALSA PCR reagents from the kit and PCR reaction was carried out by mixing 10μl of ligation product with the PCR master mix in new tubes at temperatures recommended in the protocol. All reactions were carried according to manufacturer’s protocol. The amplified PCR product was mixed with formamide, CEQ-600 marker, and 3μl of this MLPA PCR sample was then added to each well of a 96 well plate and a drop of mineral oil was added on top. Fragment separation was carried out by loading the plate into the CEQ8000 Genetic Analysis System according to manufacturer’s protocol. CSV files generated from these runs were then imported into the Coffalyser Software (MRC Holland, Amsterdam, Netherlands) for MLPA analysis.

### MSI fragment analysis

DNA was extracted from 116 macro-dissected colorectal tumors and MSI fragment analysis was performed on them and their matching normal using MSI analysis system version 1.2 kit (Promega Corporation, WI, USA). Powerplex Matrix Standards 3100/3130 kit (Promega Corporation, WI, USA) was used to perform spectral calibration of the Applied Biosystems 3130 Genetic Analyzer. The system allowed co-amplification of a total of seven markers including mononucleotide repeat markers (Bat-25, BAT-26, NR-21, NR-24 and MONO-27) and pentanucleotide repeat markers (Penta C and Penta D). MSI status was determined using the mononucleotide markers by comparing results from tumor and its matching normal, a cancer was classified as MSS when no length variations were detected between the samples for all the markers. The cancer was classified as MSI-high when 2 or more markers showed length variations between the tumors sample and its matching normal. We further classified each cancer into MSI or MSS subclasses by analyzing the expression of MLH1, MSH2, PMS2 and MSH6 using immunohistochemistry. Statistical correlation was performed for samples were both methods were used to determine MSI status.

## Results and discussion

### Genetic aberration in colorectal cancer detected using high resolution oligo-microarrays

We have performed aCGH on a total of 150 cases of CRC out of which 116 yielded acceptable DLR Spread value below 0.5 and were utilized for further analysis. Table 1 shows the clinical characteristics of the cases that were utilized for aCGH analysis.

**Table 1:** An overview of the clinical characteristics of the patients used in this study.

Patients’ characteristics	Number (Percentage)
Number of patients	
Sex	116 (100)
Male	57 (49)
Female	59 (51)
Localization	
Right	28 (24.1)
Left	47 (40.5)
Rectum	23 (19.8)
Colon unspecified	18 (15.5)
T-stage	
T-3	62 (53.4)
T-4	25 (21.6)
Unknown	29 (25.0)
Differentiation	
Well	13 (11.2)
Moderate	84 (72.4)
Poor	11 (9.5)
Unknown	8 (6.9)
Dukes’ stage	
Dukes’ B	96 (82.8)
Dukes’ C	18 (15.5)
Dukes’ D	2 (1.7)
Follow-up	
Relapsed Metastasis	11 (9.5)
Local	13 (11.2)
Disease Free	73 (62.9)
Unknown	19 (16.4)
MSI Status	
MSI	18 (15.5)
MSS	90 (77.6)
Unknown	8 (6.9)
Nationality	
West Asian	37 (31.9)
European	79 (68.1)

**Fig 1:**
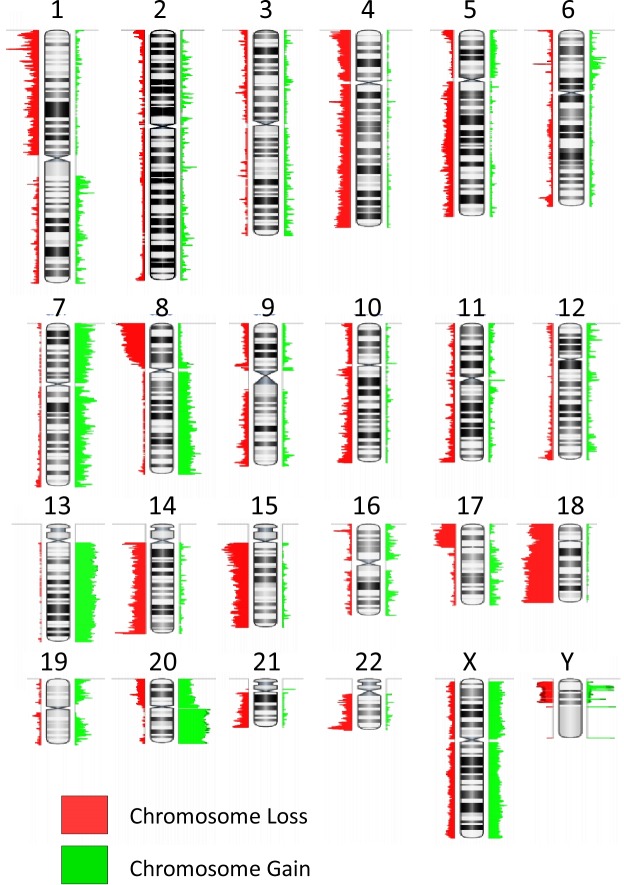
Representative karyotype colorectal cancer as determined by the Agilent Human Genome CGH Microarray 244A platform, showing summary results for those chromosome gains/losses more frequently detected in all colorectal cancer samples analyzed (n = 116). The red regions indicate loss while the green signify chromosomal gains.

The resultant aCGH profiles showed many chromosomal gains and deletions. The most frequent chromosomes involved in copy number gains in colorectal cancer were: Chromosomes 7 (56%), 8q (56%), 13 (61%), and 20 (79%). Chromosomal losses most frequently involved were chromosome arm 1p (71%), 8p (72%), 17p (55%), 22q (60%) and chromosomes 14 (77%), 15 (66%) and 18 (80%) (Fig 1). This data is consistent with previously published data from our and other groups [28–34]. However, these data represent a summation of all aberrations involved in colorectal cancer regardless of the type, stage, location and other molecular characteristics that may have a significant impact on the aCGH data obtained. Next, we subdivided this heterogeneous colorectal cancer set into genetically and phenotypically well-characterized sets and analyzed the copy number aberrations for each set using the GISTIC algorithm to obtain a functional list of genes involved in the aberrations within different subsets. It is worthy to note that our cohort was selectively biased towards TNM stage II (Dukes' B). Therefore, the frequencies of MSI and stage do not reflect population incidence. Microsatellite instability data was successfully obtained from 108 of 116 CRC cases (93%). Table 2 shows the clinico-pathological characteristics of MSS and MSI CRC cases. As expected, most MSI cases were associated with poor cancer differentiation and right sidedness.

**Table 2:** Association between clinic-pathological characteristics and MSI status.

	No. of Samples	MSS		MSI		p-Value
		No.	%	No.	%	
**Gender**						0.44
Female	54	46	51.7	8	42.1	
Male	54	43	48.3	11	57.9	
Total	108	89	100	19	100	
**Localization**						<0.0001
Left	39	38	51.4	1	6.7	
Right	26	13	17.8	13	81.3	
Rectum	20	20	27	0	0	
Transverse	4	2	2.7	2	13.3	
Total	89	73	100	16	100	
**T-stage**						0.417
T-3	56	50	72.5	6	60	
T-4	23	19	27.5	4	40	
Total	79	69	100	10	100	
**Differentiation**						<0.0001
Poor	11	4	4.8	7	38.9	
Moderate	79	68	81	11	61.1	
Well	12	12	14.3	0	0	
Total	102	84	100	18	100	
**Dukes’ Stage**						0.65
Dukes’ B	89	74	83.1	15	78.9	
Dukes’ C	17	13	14.6	4	21.1	
Dukes’ D	2	2	2.2	0	0	
Total	108	89	100	19	100	
**Follow Up**						0.27
Relapse Metastatic	9	9	11.7	0	0	
Local	12	11	14.3	1	7.1	
Disease Free	70	57	74	13	92.9	
Total	91	77	100	14	100	

### Genomic landscape patterns in MSI and MSS CRC

**Fig 2:**
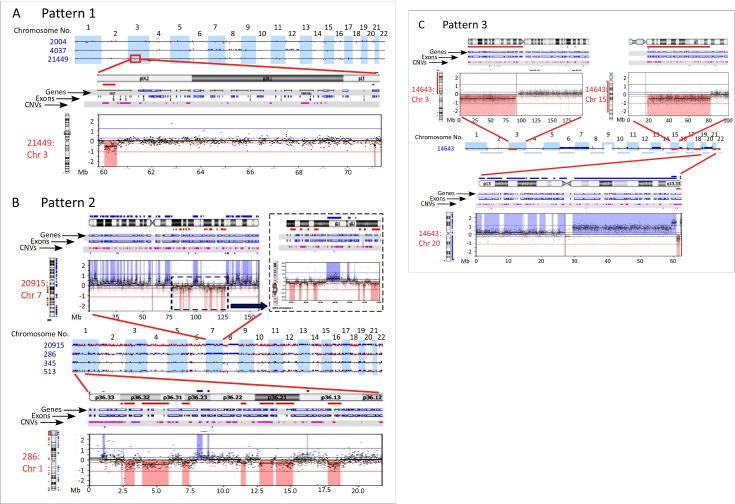
Patterns of genomic aberrations highlighted by high-resolution aCGH in CRC. a) Shows pattern 1 from 3 patients as examples (upper 22 autosomes). The zoomed inset focused on an area of chromosome arm 3P with 2 small deletions. b) Shows 4 cases of CRC with pattern 2. Notice the large number of small aberrations that alternate between gain-baseline-loss. Insets show zoomed in picture of chromosome 7 from one patients (upper panels) and chromosome 1 from a different case. Notice how well-demarcated and clear are these alternating aberrations. c) Shows a case with pattern 3, which involves copy number alterations of chromosomal arms or whole chromosomes (Insets). In all figures deletions are shown in red and deviate below the 0 (log2 ratio) and gains are depicted in blue.

Subdivision of aCGH profiles was performed to delineate distinct CNAs pertinent to the evolution of gentically and phenotypically distinct CRC subsets. Microarray data revealed 3 distinct patterns of genomic aberrations in CRC (Table 3). Pattern 1, characterized by small-sized and few gains and losses copy number alterations, was observed in 52.6% of MSI cases, and in 10.1% of MSS cases (Fig 2A). CRC with Pattern 2, on the contrary, have numerous losses and gains that cyclically alternate resembling a pattern previously described in solid tumors termed Chromothripsis [25–38]. Recently, using genome-wide long mate-pair sequencing and SNP microarray of CRC and their metastases, chromothripsis rearrangements have been found to occur frequently in CRC [39–41]. Since aCGH can only detect copy number alterations, and only that, we are not sure that these rearrangements represent true chromothripsis events. We, therefore, will refer to this pattern as chromothripsis-like or alternating copy state (Fig 2B). Chromothripsis-like events were observed in 31.6% of MSI CRC cases. In MSS CRC cases, chromothripsis-like events were uncommon and documented in only 10% of the cases. This data indicated that chromothripsis-like events were more significantly associated with MSI than MSS genotypes (p = 0.024). Pattern 3, exemplified by large sized gains and losses, which may span chromosomal arms or whole chromosomes (Fig 2C) were found in 15.8% of MSI CRC cases. As expected, this particular pattern was the most common in the genomic landscape of MSS CRC cases (89.9%).

**Table 3:** Number of aberrations among different patterns of genomic instability.

		N	Mean number of aberrations	Std. Error	95% Confidence Interval for Mean		Minimum	Maximum
					Lower Bound	Upper Bound		
Copy gain	MSS Pattern 3	80	95.90	7.945	80.09	111.71	0	358
	MSI_Pattern 1	10	34.40	11.736	7.85	60.95	4	118
	MSI_Pattern 2	6	341.83	51.592	209.21	474.45	125	458
	MSI_pattern 3	3	102.00	30.265	28.22	232.22	44	146
	MSS_Pattern 2	9	501.78	63.395	355.59	647.97	202	867
	Total	108	137.86	14.647	108.82	166.90	0	867
	Anova p value		**0.0001**					
Copy loss	MSS Pattern 3	80	115.01	9.900	95.31	134.72	4	431
	MSI_Pattern 1	10	23.50	4.636	13.01	33.99	5	56
	MSI_Pattern 2	6	277.33	60.759	121.15	433.52	96	521
	MSI_pattern 3	3	63.33	20.301	24.01	150.68	36	103
	MSS_Pattern 2	9	410.44	60.956	269.88	551.01	123	607
	Total	108	138.74	13.079	112.81	164.67	4	607
	Anova p value		**0.0001**					

### Enumeration of genomic aberrations in MSI and MSS CRC

**Fig 3:**
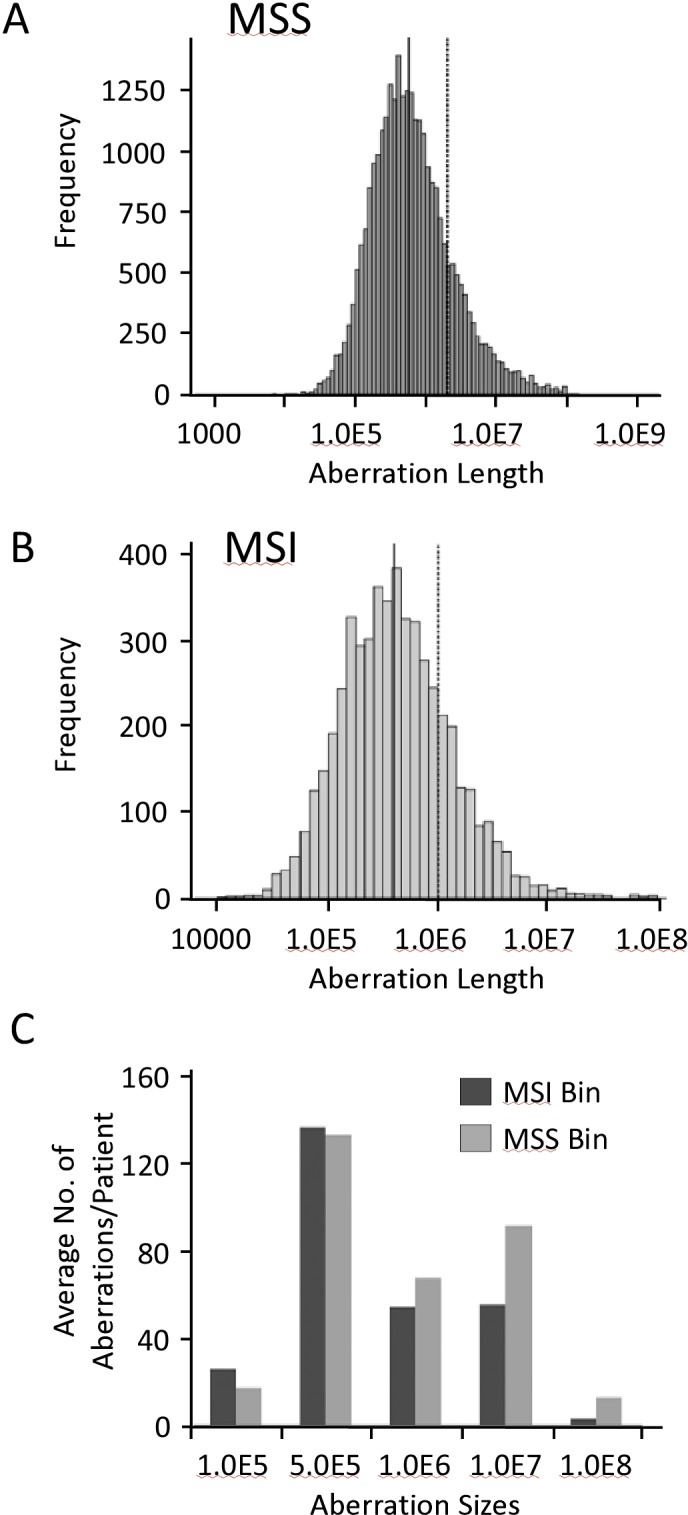
Aberrational frequencies in MSS (a) and MSI (b) CRC. The aberrational length X-axis in a and b are log transformed, with median shown as solid line and mean as dashed line. c) Shows the average number of genomic aberrations per patient in MSI and MSS CRC.

It is widely accepted that MSS CRC have higher number and larger-sized genomic aberrations compared to MSI CRC. Nevertheless, seldom do researchers divide the genomic profiles obtained by microarrays or other advanced methodologies into patterns before comparing aberration frequencies and sizes between the two groups. Fig 3 shows the frequencies of total aberrations in different classes and patterns of CRC. Table 3 subdivides these aberrations into losses and gains. As shown, the mean number of autosomal genomic aberrations in MSS CRC was 217.5 (95% C.I: 186–249), which was significantly higher than MSI CRC with pattern 1 (mean of 60 and 95% C.I: 26–95; p<0.0001) but equivalent to MSI CRC with pattern 3 genomic aberrations (mean of 170 and 95% C.I: 9.8–331.5; p>0.05). However, MSI CRC with pattern 2 had significantly more cumulative genomic aberrations than MSS CRC (mean of 624 and 95% C.I: 355–894; p<0.0001). Similarly, MSS CRC with pattern 2 had the highest number of genomic aberration (Table 3).

We next assessed and compared the length of genomic aberrations in MSS and MSI CRC. Fig 3 shows the histogram spread of genomic aberration sizes with the indicated median and mean length in MSS (Fig 3A) and MSI (Fig 3B) CRC. On average, patients with MSI CRC had smaller sized genomic aberrations compared to MSS CRC patients (Fig 3C).

### Unsupervised clustering of genomic aberrations in CRC

**Fig 4:**
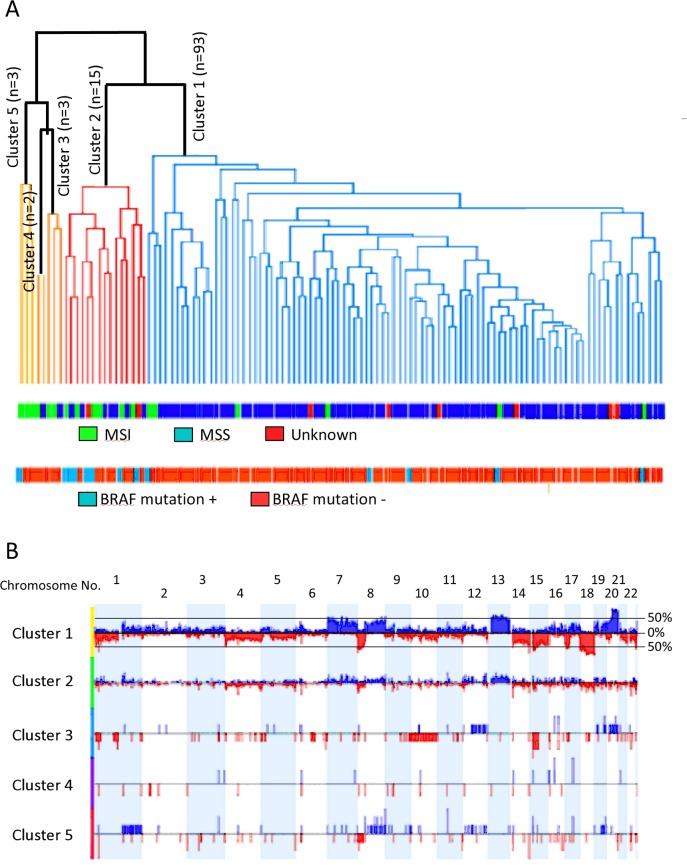
a) Complete Linkage Hierarchical clustering of 116 cases with CRC. The generated 5-clusters are depicted in the dendogram. The lower panels show microsatellite stability and BRAF gene status. b) Frequency (aggregate) histograms from 5-groups of CRCs clustered according to copy number aberration patterns. The upper histogram represents the summation of all the aberrations in 116 cases of CRC. Deviations up from 0% (Log2 ratio) represent copy number gains and are colored blue, while deviations below the 0% line (red) represent copy number losses. The y-axis represents % of samples with a specific aberration. The two horizontal lines demarcate the cutoff (35%) used to call aberrations in the STAC algorithm. The lower histograms are aberrations found in the 5 generated clusters.

We employed unsupervised complete Linkage Hierarchical clustering on the 116 CRC cases. The method orders cases based solely on their genomic aberrations without prior knowledge of other clinical, genetic or MMR data. Fig 4A shows the 5 clusters generated from this analysis. The CNAs of each group/cluster are shown in Fig 4B. Cluster 1 (group 1) identified genomic aberrations significantly associated with MSS CRC harboring largely wild type *BRAF*, and were left-sided with rectosigmoidal subsite enrichment (Table 4).

**Table 4:** Clinicopathological characteristics of the 5-clusters generated by unsupervised complete Linkage Hierarchical clustering.

	Group 1	Group 2	Group 3	Group 4	Group 5	
	N = 93 (p value)	N = 15 (p value)	N = 3 (p value)	N = 2	N = 3 (p value)	P value between groups 1 and 2
**Clinical characteristics**						
**Microsatellite**						
Stable (MSS) n = 89	81(1.2×10^−6^)	6	1	1	0	
Instable (MSI) n = 19	6	7(0.003)	2(0.06)	1	3(0.003)	p0.00013
MMR unknown n = 8	6	2				
**BRAF mutation**						
Negative n = 103	88(5.1×10^−4^)	8	3	2	2	
Positive n = 13	5	7(1.5×10^−4^)	0	0	1	p<0.0001
KRAS mutation						
Negative n = 88	75(0.0188)	7	2	2	2	
Positive n = 20	16	2	1	0	1	
**CRC site**						
Left n = 67	62(1.1×10^−4^)	4	0	1	0	
Right n = 31	17	8(0.018)	2	1	3(0.018)	0.003
**CRC subsite**						
Rectum n = 23	21	2	0	0	0	
Rectosigmoid n = 13	13(0.047)	0	0	0	0	
Descending/Sigmoid n = 28	25	2	0	1	0	
Transverse colon n = 4	2	1	0	0	1	
Ascending n = 13	6	5(0.003)	0	0	2(0.019)	
Caecum n = 14	9	2	2(0.038)	1	0	
**Survival**						
Disease-recurrence n = 24	23(0.022)	0	0	0	1	0.18
Disease-free n = 63	51	7	1	2	2	

We next employed a global frequency statistical approach using the significance testing for aberrant copy number (STAC) algorithm. STAC-based algorithm is a robust method which identifies a set of aberrations that are stacked on top of each other from different patients or microarrays such that it would not occur by chance. To find these events, aberrations (usually narrow regions) are permuted in each arm of each chromosome and assess how likely it is for an event to occur at any location at a particular frequency set here at 35% and minimum p<0.05 [39]. Cluster 1, grouped samples with broad high frequency gains in chromosome arms 20q (58–97%, p<0.0001), 13q (63–71%, p<0.0001), 7 (41–63%, p<0.0001), 8q (45–62%, p<0.0001), and broad losses in chromosomes 18 (76–91%, p<0.0001), 14q (45–84%, p<0.0001), 8p (65–82%, p<0.0001), 15q (54–76%, p<0.0001), and 1 (35–76%, p<0.0001). Cluster 2, identified cases with MSI, which also harbored *BRAF* V600E mutation, were significantly associated with right-sidedness and ascending colon subsite enriched (Table 4). This cluster had low frequency gains and losses stretching along the genome, with specific and significant peaks of gains at chromosome arms 1q 23.3 (40%, p<0.0001), 11q11 (40%, p<0.0001), 12q24.31 (40%, p<0.001), 13q14.12–14.13 (40%, p<0.005), and losses at chromosome arms 14q11.2 (60%, p<0.0001), 22q11.23 (53%, p<0.0001), 6q21.32 (47%, p<0.0001), 14q32.33 (40%, p<0.01), 16p13.2 (40%, p<0.0001), 8p23.3, 8p23.1,18q12.2 and 18q23 (40%, p<0.02). The other clusters, namely clusters 3, 4 and 5, aggregated small number of cases and exhibited similar characteristics to group 2 (Table 4). Group5, specifically, was similar to group 2, while group 3 was enriched with cecal cancers.

### Genomic aberrations in MSI and MSS CRC using supervised clustering analysis

**Fig 5:**
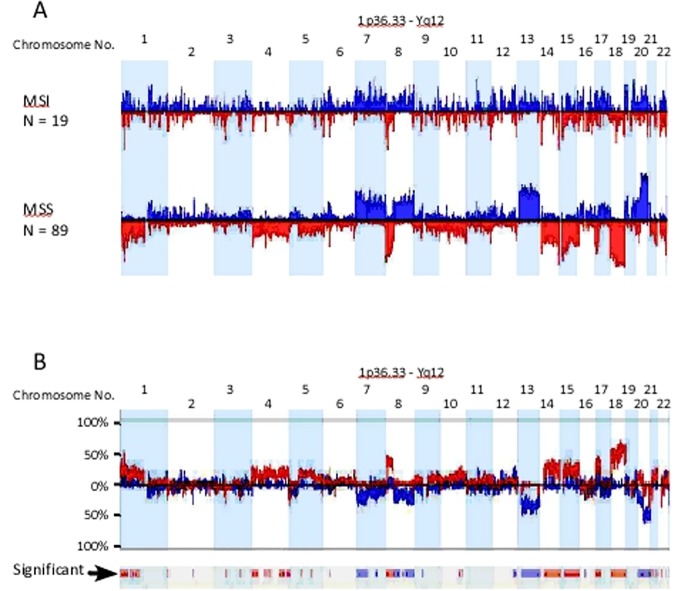
Genomic aberrational differences between MSS and MSI CRC. a) Shows histogram profiles of the autosome from 19 MSI and 89 MSS CRC. Deviations up from 0% represent copy number gains and are colored blue, while deviations below the 0% line represent copy number losses and are colored red. b) Genomic aberrations frequency (or %) differences between MSS and MSI CRC. The significant differences are displayed as bars in the significance row marked with black arrow. Blue bars deviating below 0% indicates significantly more frequent gains in MSS compared to MSI CRC, while blue bars deviating above 0% represent more frequent gains in MSI compared to MSS (e.g. chromosome arm 12q). Similarly, red bars deviating above 0% indicates significantly more frequent losses in MSS compared to MSI CRC, while red bars deviating below 0% represent more frequent losses in MSI compared to MSS (e.g. chromosome 3p).

Genetic aberrations generated by Supervised clustering from well-defined sets of samples, in this case 19 and 89 cases of MSI and MSS CRC respectively, are shown in (Fig 5A). Consistent with unsupervised clustering, the genomic aberration profiles of MSS and MSI CRC were largely identical to group 1 and group 2 respectively (Fig 5A). Direct comparison of the generated genomic profiles between MSS and MSI CRC, highlighted the extensive differences in genomic aberrations between the two groups (Fig 5B). Chromosome arms 1p, 8p, 10q, 17p and chromosomes 4, 14, 15, 18 deletions were significantly more frequent in MSS than MSI CRC (Table 5). Similarly, chromosome arms 7p, 8q, 12q, 20q and chromosome 13 gains were more frequent in MSS compared to MSI CRC (Fig 5B and Table 6). Other small aberrations on chromosomes 3p, 5, 21 and 22 were interesting. For example, the 3p small deletion was a common event (33%) in our CRC cases (Fig 5A and 5B). The deletions were well demarcated around exon 5 of the *FHIT* gene and appeared in both MSS and MSI CRC, although more significantly deleted in MSI cancers (Fig 5B). *FHIT* gene deletion and its reduced expression have been reported before in association with MSI CRC by our group [42].

**Table 5:** Copy number aberrations generated by the GISTIC algorithm in MSS CRC.

Narrow Region*	Extended Region	Type	Q-Bound	G-Score
chr22:22,669,244–22,674,560	chr22:22,669,244–22,686,007	Loss	2.54E-10	61.1
chr6:32,563,052–32,576,786	chr6:32,563,052–32,603,160	Loss	2.54E-10	58.3
chr4:69,305,945–69,550,743	chr4:69,295,525–69,550,743	Loss	2.54E-10	56.7
chr20:47,701,770–47,747,973	chr20:47,664,076–49,743,884	Gain	3.06E-10	55.6
chr8:39,386,202–39,461,142	chr8:39,365,809–39,496,849	Loss	2.54E-10	48.9
chr18:74,434,936–74,528,730	chr18:72,984,865–76,117,153	Loss	2.54E-10	41.0
chr13:72,532,072–72,545,802	chr13:72,468,464–72,879,517	Gain	3.06E-10	34.1
chr15:19,938,622–19,973,512	chr15:19,884,805–19,973,512	Loss	2.54E-10	33.9
chr3:163,992,380–164,107,362	chr3:163,992,380–164,107,362	Loss	2.54E-10	33.7
chr14:105,283,523–105,311,171	chr14:105,151,893–105,479,165	Loss	2.54E-10	32.1
chr20:25,853,011–25,926,849	chr20:25,830,521–27,100,000	Gain	3.06E-10	31.8
chr8:39,373,309–39,485,162	chr8:39,373,309–39,491,113	Gain	3.06E-10	30.7
chr8:126,438,529–126,498,088	chr8:123,842,688–126,506,271	Gain	3.06E-10	29.6
chr1:149,376,254–149,397,162	chr1:149,367,733–149,397,162	Loss	2.54E-10	28.7
chr1:18,205,194–18,286,328	chr1:18,197,094–18,317,078	Loss	2.54E-10	28.6
chr8:1,059,261–1,099,327	chr8:0–1,321,118	Loss	2.54E-10	28.0
chr17:11,096,055–11,171,903	chr17:9,647,537–13,915,524	Loss	2.54E-10	26.8
chr7:27,449,750–27,595,647	chr7:26,477,611–30,369,308	Gain	3.06E-10	25.3
chr16:6,504,522–6,512,278	chr16:6,443,207–6,531,574	Loss	2.54E-10	24.6
chr11:55,191,165–55,197,896	chr11:55,191,165–55,216,250	Loss	2.54E-10	23.8
chr11:55,131,937–55,191,165	chr11:55,115,301–55,191,165	Gain	6.85E-10	23.5
chr5:180,359,104–180,364,172	chr5:180,268,615–180,364,172	Loss	2.54E-10	22.9
chr7:75,046,728–75,065,482	chr7:73,034,227–75,365,689	Gain	3.91E-09	22.4
chr6:162,739,756–162,757,660	chr6:162,543,026–163,000,404	Loss	2.54E-10	22.1
chr20:14,855,016–14,880,574	chr20:14,752,042–14,916,356	Loss	2.61E-10	21.7
chr4:1,372,689–1,433,700	chr4:979,401–1,522,379	Loss	2.61E-10	21.6
chr2:132,598,122–132,890,744	chr2:132,598,122–132,900,372	Gain	2.26E-07	20.2
chr19:18,362,768–18,392,988	chr19:17,152,138–18,542,288	Gain	6.37E-07	19.6
chr21:46,806,563–46,851,646	chr21:46,023,979–46,851,646	Loss	6.36E-09	19.1
chr17:41,572,059–41,640,287	chr17:41,497,558–41,672,900	Gain	3.17E-06	18.6
chr1:146,605,059–146,672,075	chr1:146,605,059–146,686,184	Gain	4.87E-06	18.3
chr12:15,738,418–15,751,788	chr12:15,710,138–15,827,008	Gain	6.97E-06	18.1
chr10:38,700,181–39,018,864	chr10:38,700,181–39,018,864	Gain	8.02E-06	18.0
chr16:29,840,265–29,885,801	chr16:29,566,294–30,389,686	Gain	1.51E-05	17.6
chr19:53,811,858–53,897,694	chr19:53,290,145–54,363,641	Gain	2.01E-05	17.4
chr21:9,896,630–10,038,957	chr21:9,896,630–10,065,864	Gain	2.05E-05	17.4
chr10:134,586,239–134,803,162	chr10:134,298,652–134,938,121	Loss	3.91E-07	17.2
chr6:26,352,706–26,361,349	chr6:26,256,770–26,720,741	Gain	4.04E-05	16.9
chr14:19,417,751–19,449,642	chr14:19,278,801–19,493,856	Loss	1.51E-06	16.6
chr12:130,314,128–130,348,539	chr12:130,249,201–130,408,894	Loss	1.77E-06	16.5
chr9:45,357,769–45,739,136	chr9:45,357,769–45,739,136	Gain	1.12E-04	16.2
chr1:145,917,263–146,000,447	chr1:145,885,332–146,000,447	Loss	2.83E-05	15.1
chr3:163,992,380–164,107,362	chr3:163,992,380–164,107,362	Gain	0.0012065	14.5
chr3:60,454,798–60,465,650	chr3:60,383,448–60,485,055	Loss	1.04E-04	14.4
chr9:135,119,951–135,258,527	chr9:134,748,046–135,386,643	Loss	1.59E-04	14.1
chr4:69,218,552–69,295,525	chr4:69,127,903–69,295,525	Gain	0.0032968	13.8
chr22:47,396,201–47,433,182	chr22:47,327,520–47,701,683	Loss	5.73E-04	13.4
chr9:40,501,951–41,897,530	chr9:39,152,128–41,897,530	Loss	5.92E-04	13.4
chr11:131,509,992–131,674,900	chr11:131,491,803–133,419,821	Loss	7.30E-04	13.3
chr1:0–738,061	chr1:0–738,061	Gain	0.0068923	13.2
chr22:22,717,333–22,722,828	chr22:22,717,333–22,722,828	Gain	0.0082828	13.0
chr5:732,646–742,598	chr5:0–754,698	Loss	0.0020752	12.7
chr16:67,887,820–68,061,099	chr16:65,245,773–68,673,221	Gain	0.0157484	12.5
chr6:29,956,856–30,007,461	chr6:29,956,856–30,016,093	Loss	0.0030972	12.4
chr10:1,207,969–1,325,890	chr10:0–3,675,211	Loss	0.0039758	12.2
chr2:61,332,414–61,586,388	chr2:61,102,731–65,541,488	Gain	0.0241268	12.1
chr16:54,349,083–54,384,952	chr16:54,349,083–54,384,952	Loss	0.0060993	12.0
chr9:42,913,008–43,080,383	chr9:42,913,008–43,080,383	Loss	0.0063739	11.9
chr18:1,610,018–1,759,320	chr18:917,185–2,088,004	Loss	0.0082034	11.8
chr4:191,070,477–191,128,555	chr4:191,054,399–191,128,555	Loss	0.0094985	11.7
chr15:94,744,405–94,838,744	chr15:92,681,164–96,935,377	Loss	0.0395216	10.7
chr11:5,740,514–5,764,428	chr11:4,333,855–6,580,666	Loss	0.0395216	10.7

*Shaded rows identify regions common to MSI CRC.

**Table 6:** Copy number aberrations generated by the GISTIC algorithm in MSI CRC.

Narrow Region*	Extended Region	Type	Q-Bound	G-Score
chr6:32,576,786–32,603,160	chr6:32,563,052–32,644,260	Loss	4.82E-12	12.7
chr8:39,349,089–39,359,852	chr8:39,349,089–39,365,809	Loss	8.29E-09	10.7
chr22:22,669,244–22,717,333	chr22:22,645,114–22,722,828	Loss	1.42E-06	8.5
chr14:19,487,351–19,493,856	chr14:18,753,436–19,493,856	Loss	5.47E-06	7.9
chr1:149,367,733–149,397,162	chr1:149,367,733–149,397,162	Loss	6.95E-06	7.8
chr1:193,475,381–193,529,957	chr1:193,456,932–193,544,824	Loss	4.26E-05	7.2
chr14:105,589,128–105,636,720	chr14:105,589,128–105,636,720	Loss	4.26E-05	7.2
chr11:55,115,301–55,191,165	chr11:55,115,301–55,216,250	Gain	0.002372	7.1
chr15:19,501,226–19,796,337	chr15:19,351,570–20,150,205	Loss	4.26E-05	7.1
chr3:60,339,210–60,409,611	chr3:60,339,210–60,731,181	Loss	1.92E-04	6.6
chr8:7,258,853–7,953,633	chr8:267,005–8,144,283	Loss	2.44E-04	6.5
chr6:245,716–327,686	chr6:245,716–327,686	Loss	5.65E-04	6.0
chr4:69,295,525–69,550,743	chr4:69,127,903–69,550,743	Loss	0.001436	5.5
chr1:18,197,094–18,292,586	chr1:18,175,101–18,292,586	Loss	0.001436	5.5
chr18:33,269,571–33,343,946	chr18:31,514,045–42,919,686	Loss	0.00465	4.8
chr11:134,210,403–134,452,384	chr11:133,657,612–134,452,384	Loss	0.011382	4.4
chr16:54,349,083–54,384,952	chr16:52,708,563–54,384,952	Loss	0.012709	4.3
chr21:13,591,226–14,355,692	chr21:13,572,244–14,355,692	Loss	0.015794	4.1
chr12:130,314,128–130,408,894	chr12:129,946,814–130,706,577	Loss	0.018008	4.1
chr7:141,962,422–141,981,657	chr7:141,148,142–158,628,139	Loss	0.019226	4.0
chr9:42,913,008–43,080,383	chr9:42,913,008–43,080,383	Loss	0.019226	4.0
chr20:57,698,990–57,804,320	chr20:57,664,611–57,848,527	Loss	0.027869	3.8
chr11:5,735,996–5,764,428	chr11:4,455,169–6,584,397	Loss	0.035934	3.7
chr16:34,318,531–34,455,993	chr16:34,148,509–35,005,009	Loss	0.040215	3.7
chr22:46,946,978–47,684,217	chr22:46,946,978–47,900,828	Loss	0.049436	3.5

*Shaded rows point to deletions private to MSI and not identified by the GISTIC algorithm as significant in the MSS CRC series.

### Functional relevance of genomic aberrations in MSS and MSI CRC

It is well accepted that CNA in cancer involves many random genetic events that frequently distort and may conceal the important genomic aberrational events that are functionally relevant. Our classical comparison of the frequencies of genetic events between MSS and MSI CRC cases highlighted the extensive and widespread differences in genomic aberrations between the two groups (Fig 5B). However, how 'genomically' close or apart are MSS and MSI CRC in terms of functional or driver mutations, is a question that has not been well-addressed before. Therefore, we next reanalyzed the 108 dataset using 2 independent algorithms that focus on the functional relevance of a particular genomic aberration. First, we used STAC algorithm approach to identify statistically significant and recurrent genomic CNAs within subsets of samples under the same settings mentioned above. The second algorithm GISTIC, which identifies functionally significant CNAs by giving more weight to high copy gains and homozygous losses (amplitudes), which may be functionally relevant to the successful evolution of the cancer genome [27]. Such permutations are not considered in the STAC analysis we used. Therefore, the two methods may be considered complementary rather than comparable.

**Fig 6:**
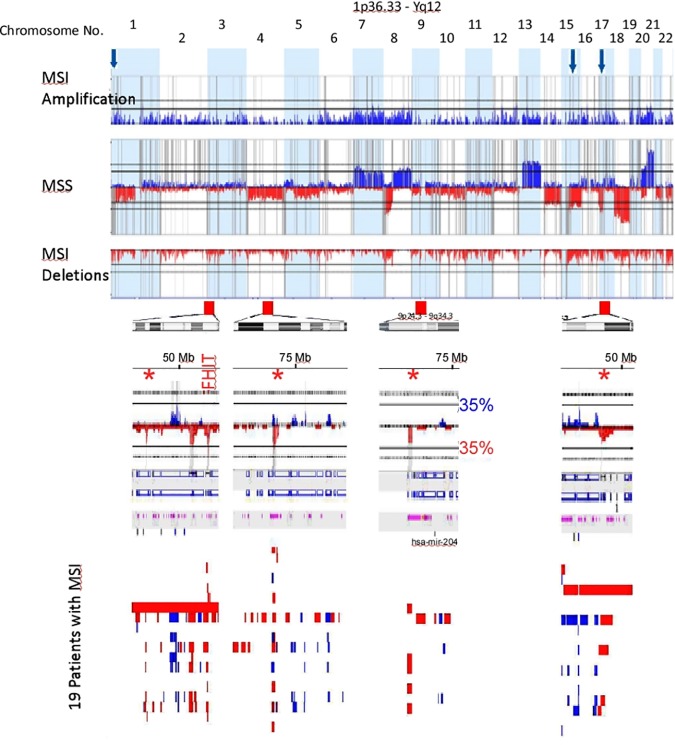
Frequency profiles of copy number aberrations from 89 MSS (middle histogram; with gains above 0% line shown as blue peaks and below 0% as deletions in red) and 19 MSI CRC cases (divided histogram; upper showing gains in blue and lower histogram shows deletions in red). The vertical grey lines indicate significant peaks of copy number alterations. Notice how these peaks align precisely in both MSS and MSI CRC. Copy number aberrations that are private for MSI CRC cases are marked with red arrows for deletions and blue arrows for amplifications. Below each red arrow are the corresponding chromosomal region ideogram and a zoomed histogram focused on the red arrowed region. The histograms show deletions in red and amplifications in blue and significant STAC-generated peaks in grey as stated above. Deletions marked with asterisks correspond to the deletions marked with the red arrows. All these aberrations are not considered in the exported regions because they do not reach the 35% cutoff value (marked horizontal black lines on chromosome 9). Note how the marked FHIT deletion crosses the 35% line. The individual copy number alteration for each patient from 19 MSI cases are shown as small vertical red and blue bars indicating deletions and amplifications respectively. Note how well-demarcated are these recurrent aberrations.

Fig 6 shows functionally significant CNAs generated by the STAC algorithm in MSI and MSS CRC. The peaks identify aberrations that are significantly recurrent. As demonstrated before with the traditional frequency-based method, that MSS CRC accumulated more CNAs than MSI CRC, albeit, more functionally relevant here. A remarkable observation that emanated from the use of the STAC algorithm was the high-degree of similarity between the functionally-relevant copy number alterations in MSI and MSS CRC. On almost all the autosomes, functionally significant CNAs found in MSI CRC mapped precisely onto those found in MSS CRC (Fig 6). All genomic aberrations specific to MSI CRC are highlighted with arrows in Fig 6. These aberrations did not reach the 35% cutoff, or did not contain functional genes (chromosome 4) and therefore excluded by the algorithm.

**Fig 7:**
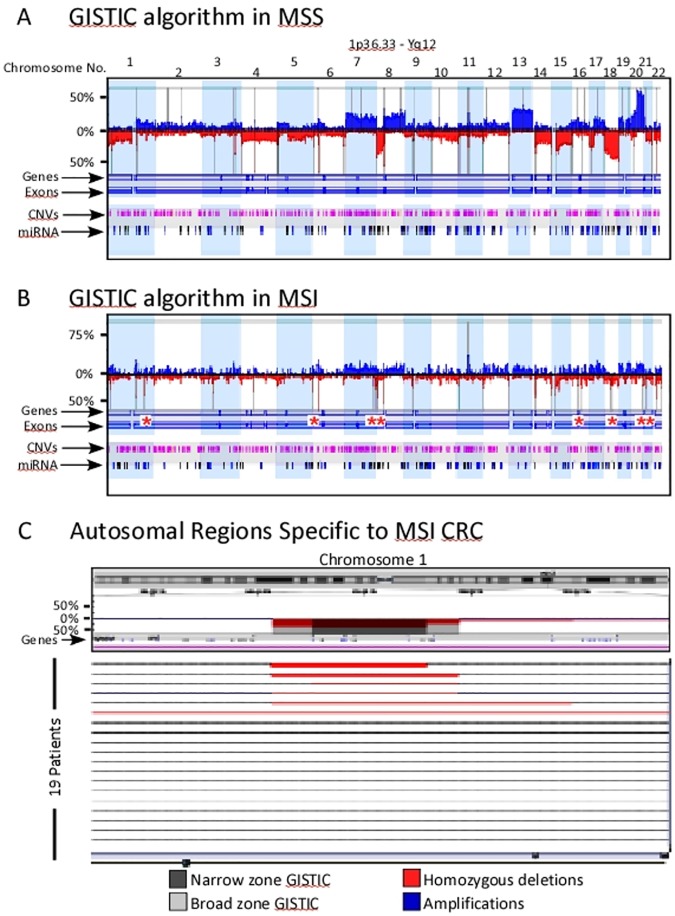
GISTIC analysis of MSS and MSI CRC copy number aberrations. a) Histogram profile of the autosome from 89 MSS and b) from 19 MSI CRC. Deviations up from 0% represent copy number gains and are colored blue, while deviations below the 0% line represent copy number losses and are colored red. Vertical grey lines indicate regions identified as significant using the Q-Bound (p>0.05) and G-score>1. Asterisks, pinpoint narrow GISTIC regions identified only in MSI CRC cases. c) Zoomed in view of the regions private to MSI CRC showing the corresponding chromosome ideogram on top and focused GISTIC-identified areas with broad (grey) and narrow (dark grey) zones along the histograms (middle) and the 19 patients (lower panels), each represented in a horizontal line indicating the deletions in red, homozygous deletions in dark red/broad red lines and amplifications in blue.

We next employed the GISTIC algorithm on the same data set. Fig 7 shows the significant CNAs in both MSS (Fig 7A) and MSI (Fig 7B) as broad and narrow regions represented by vertical grey lines. Again, these functionally significant aberrations were more common in MSS compared to MSI CRC cases (Tables 5 and 6). Genes involved in cellular senescence, S-phase control of the cell cycle, histone H4-K5 acetylation, nucleosome assembly and telomere maintenance were among the top 10 most significant GO-functions identified.

Deletions were the most overwhelming functional aberrations identified by the GISTIC algorithm in MSI CRC (Fig 7B). As proposed by the STAC algorithm, the GISTIC-generated regions in MSI corresponded precisely to regions on the MSS histogram (Fig 7 and Table 5). However, GISTIC analysis identified 8 narrow autosomal regions specific to MSI CRC (Fig 7B and 7C and Table 6).

**Fig 8:**
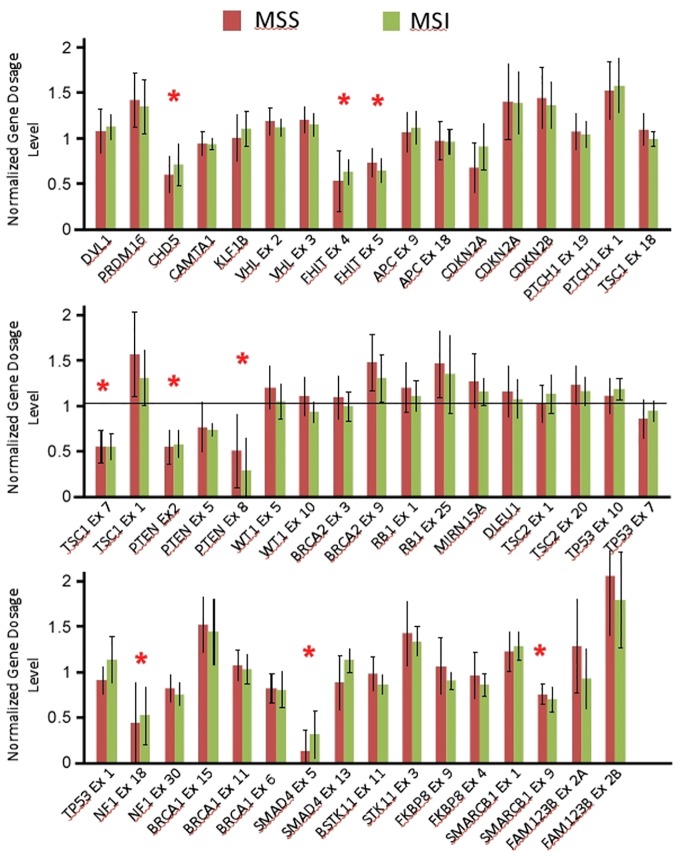
Normalized gene dosage levels (Y-axis) for 20 tumor suppressor genes (X-axis) generated using MLPA. Asterisks indicate deleted genes/exons.

Our STAC profiling suggests, with the exception of 8 private genomic copy alterations identified by the GISTIC algorithm, that MSI and MSS CRC functional CNAs are similar. These shared genomic events may be key in the evolution of both types of CRC. These reserved CNAs might be critical to the oncogenesis in primary cellular transformation stages, later distinct CNAs specific to MSI and MSS might occur due to the influence of specific tumor microenvironments. For example, it has been shown that MSI distinct CNAs include immune related genes that alters their susceptibility to the immune system, hence MSI CRC’s favorable prognosis [43–44]. Regardless of the mechanism of how such similarities in CNAs are attained in these two distinct groups, our data argue that, at least at the genomic copy number level, the evolutionary and biologically relevant driver mutations of MSI CRC are a subset of those found in MSS CRC, and that the two groups are not as dissimilar with respect to functional CNAs as traditional methods have previously suggested. We next tested this notion using MLPA tumor-loss panel (P294-A1), which profiles 20 key tumor suppressor genes that are frequently deleted in cancer with 50 probes spanning different exons of the corresponding genes. Due to the limited tissues available to us, MLPA was performed on microdissected DNA extracted from 44 cases (subset of 116 cases, used for aCGH). The MMR status was known for 40 cases (MSI n = 7 and MSS n = 33). Fig 8 shows the normalized copy number ratios for the 20 tumor suppressor genes distributed according to MMR status. The data show loss of 7 tumor suppressor genes in this cohort; *CHD5*, *FHIT*, *TSC1* (exon 7), *PTEN*, *NF1*, *SMAD4* (exon 5), and *SMARCB1* (exon 9) irrespective of the MMR status (Fig 8).

The genomic aberrations detected in MSS and MSI may have a direct effect on genes’ expression, which are thought to be the driving force of disease pathology of both types of tumors as suggested previously by several groups [45–48]. Kheurekseid et al., (2013) identified a comprehensive list of genes showing clear differential expression patterns in CRC. Most of these genes are located in genomic regions well affected by the aberrations found in our study. Unfortunately, we were limited by the use of FFPE tissues to pursue each of the genes located within the CNAs identified. Understanding the molecular impact of these expression alterations would provide a better understanding of the molecular pathology of CRC. Localizing the culprit genes involved in CNAs can also serve as molecular markers to aid in diagnosis, follow up and most importantly potential development of individualized therapeutic strategies. This study therefore was limited in its approach and may benefit from a direct correlation between genes located within the CNAs and their expression. Nevertheless, this study illuminated genomic deletions as a possible mechanism for loss or reduced expression of the 7 tumor suppressor genes regardless of the MMR status. The *FHIT* gene deletion was identified previously by us and others as a mechanism for the reduced FHIT protein expression in CRC [42,49]. The tumor suppressors TSC1 and PTEN protein expression were shown to be reduced in CRC compared to normal tissues or adenomas [50,51]. Similarly, SMAD4 and SMARCB1 expression were absent/reduced in 64% [52], 67% [53] of CRC respectively. Interestingly, CHD5 protein expression was repressed in the majority of adenomas via genetic deletion, hypermethylation or by microRNA-211 suppression [54,55]. Our study is supportive of genomic deletions as partly the reason behind the lack or reduced expression of these tumor suppressor genes in CRC regardless of the MMR status.

### Conclusion

The last decade witnessed a substantial increase in our understanding of the genomic landscape of CRC. The use of low resolution genome-profiling technologies, like BAC-microarrays to identify copy number differences between MSS and MSI CRCs propagated the notion that these two subtypes of CRC are substantially different at the copy number level. Our high resolution genetic profiling of CRC supports this genomic heterogeneity between MSS and MSI CRC. For example, MSS-CRC had more numerous larger aberrations while MSI-CRC had smaller-sized aberrations. Moreover, there were distinct patterns of genomic aberrations in CRC, one of which, pattern 2 (chromothripsis-like) was significantly enriched in MSI CRC, while MSS CRC was significantly associated with Pattern 3 (loss/gain of whole chromosomes or chromosome arms). In fact, the examination of the genomic histograms showed significant differences between MSI and MSS as previously reported. However, closer inspection of the patterns generated suggests that, although there were unique copy number differences, MSI and MSS CRC functional copy number aberrations are similar. These shared genomic events could highlight key events in the evolution of both types of CRC. This finding supports the hypothesis that driver mutations incurred by CNAs in MSI CRC are also found in MSS CRC, and that the two groups are not as dissimilar in respect to CRC development. Our results suggest that MSI and MSS copy number aberrations driving CRC development may be functionally comparable.

## Abbreviations

aCGH: Array-based comparative genomic hybridization
BAC: Bacterial artificial chromosome
mCGH: Metaphase-based comparative genomic hybridization
CNA: Copy number aberration
MSS: Microsatellite Stable
STAC: Significance testing for aberrant copy number
GISTIC: Genomic Identification of Significant Targets in Cancer
CRC: Colorectal Carcinomas
MSI: Microsatellite Instability
MLPA: Multiplex Ligation-dependent Probe Amplification
FFPE: Formalin-fixed paraffin-embedded
DLRS: Derivative of log ratio spread
FASST: Fast Adaptive State Segmentation Technique
